# *Lactobacillus helveticus* CNU395 and *L. paracasei* CNU396 Alleviate Cognition in Scopolamine-Induced Cognitive Impairment Mice

**DOI:** 10.3390/microorganisms13081714

**Published:** 2025-07-22

**Authors:** Bao Le, Dong Gyun Kim, Hyun Song, Phan Duy Khanh Giang, Ho Tue Han, Seung Hwan Yang

**Affiliations:** 1 Research Group in Pharmaceutical and Biomedical Sciences, Faculty of Pharmacy, Ton Duc Thang University, Ho Chi Minh City 700000, Vietnam; lebao@tdtu.edu.vn (B.L.); h2100409@student.tdtu.edu.vn (P.D.K.G.); h2100032@student.tdtu.edu.vn (H.T.H.); 2 Department of Biotechnology, Chonnam National University, Yeosu 59626, Republic of Korea; don92166@naver.com (D.G.K.); thdgus1020@naver.com (H.S.)

**Keywords:** behavioral test, cognitive impairments, Lactobacillus, probiotics, scopolamine

## Abstract

This study aimed to evaluate whether probiotic administration could protect against cognitive impairments in a scopolamine-induced cognitive impairment mice model. Male C57BL/6 mice (8 weeks of age) were injected with scopolamine hydrobromide to induce memory impairments. The experimental groups were additionally supplemented with 10^9^ colony-forming units (CFU)/day probiotics containing *Lactobacillus helveticus* CNU395 or *L. paracasei* CNU396. Behavioral test results and histopathological evaluations showed that the spatial memory ability and pathological tissue abnormalities of the mice in the CNU395 and CNU396 groups significantly improved compared with those in the disease group. CNU395 and CNU396 mitigated scopolamine-induced neuroinflammation by reducing the expression of pro-inflammatory cytokines (*IL-6*, *IL-8*, *IL-10*, and *TNF-α*) and the *NLRP3* inflammasome, through the inhibition of *MAPK* and *NF-κB* inflammatory pathways. Additionally, the CNU395 and CNU396 groups showed decreased levels of *Iba-1* and *Bax*, alongside increased levels of *BDNF* and *Bcl-2*, relative to the disease group. Therefore, CNU395 or CNU396 supplementation might help prevent the onset of cognitive deficits and neuroinflammation.

## 1. Introduction

Alzheimer’s disease (AD) is a neurodegenerative disorder that affects memory, thinking, and behavior. It is one of the leading causes of death in Vietnam and other countries [1]. It is driven by multiple factors, including amyloid beta (Aβ) accumulation, increased tau protein phosphorylation, excitotoxicity, oxidative stress, and neuroinflammation [2]. AD development is also influenced by modifiable risk factors such as smoking, physical inactivity, and diet [3]. However, evidence supporting the effectiveness of interventions that target the causes and risk factors of AD is insufficient.

Studies have demonstrated that a decrease in cholinergic neurons in the cerebral cortex and hippocampus affects AD development [4]. Cholinergic neurons secrete acetylcholine (ACh), a neurotransmitter involved in memory and learning. Therefore, acetylcholinesterase (AChE) inhibitors are considered the preferred drug class for AD treatment. AChE inhibitor prodrugs may help increase acetylcholine concentration in the synapses. However, the clinical relevance of these effects remains unclear [5]. Additionally, synthetic drugs can cause side effects, such as a loss of appetite, fatigue, diarrhea, muscle cramps, and poor sleep.

The endogenous intestinal microbiota is essential for maintaining human health. Its structure, quantity, distribution, and biological characteristics are diverse and constantly changing [6]. It participates in energy metabolism, reduces inflammatory responses, stimulates systemic immunity, protects the host from pathogen invasion and infection, and promotes nutrient absorption. Recently, probiotics have been used to treat many gastrointestinal and neurodegenerative diseases, including AD [7]. Some studies have shown that multi-strain probiotic formulations are more efficient, such as Akbari et al. [8] who evaluated the effects of a probiotic cocktail (*Lactobacillus acidophilus*, *L. casei*, *L. fermentum*, and *Bifidobacterium bifidum*) on the cognitive function and metabolic status of 60 patients with AD. After 12 weeks of treatment, the Mini-Mental State Examination scores of the 30 patients in the probiotic intervention group were higher than those in the control group. The mixture of *L. acidophilus*, *L. fermentum*, *Bifidobacterium lactis*, and *B. longum* improves learning ability and spatial memory in AD rats [9]. Additionally, a report suggests that consuming a combination of *L. helveticus* R0052 and *Bifidobacterium longum* R0175 (Probio’Stick) for one month may help reduce anger, depression, and anxiety while lowering cortisol levels [10]. However, less is known about the effect of single-strain probiotics; knowledge on its mechanism and metabolomic interaction is still lacking. In animal models, Maftoon et al. [11] found that *Akkermansia muciniphila* could influence the central nervous system through the gut–brain axis in a tau protein-induced AD rat model. Moreover, *Lactobacillus paracasei* PS23 was shown to enhance the brain-derived neurotrophic factor (BDNF), which plays an important role in neuronal survival in a chronic corticosterone-induced depression mice model [12]. Because of the variability in efficacy among bacterial strains and species, the efficacy of single probiotic strains on AD development should be further investigated.

Scopolamine induces memory and cognitive deficits in an in vivo model to study dementia-related disorders such as AD [13]. In our previous study, a *L. helveticus* CN395 fermented *Scrophularia buergeriana* extract increased the expression of *IL-6* and *TNF-α* in LPS-stimulated RAW 264.7 cells [14]. The aim of this study was to explore the potential neuroprotective efficacy of two bacterial strains (*Lactococcus helveticus* CNU395 and *L. paracasei* CNU396) on scopolamine-induced cognitive impairment in C57BL/6 mice. We aimed to investigate the effect of probiotics on behavioral characteristics and determine the mechanism underlying the improvement of cognitive symptoms. The selection of the bacterial strains was based on previous studies that highlighted their potential benefits for mental health.

## 2. Materials and Methods

### 2.1. Preparation of Probiotics

Two bacterial strains, namely, *L. helveticus* CNU395 and *L. paracasei* CNU396 are proprietary strains isolated from homemade Korean kimchi samples using a de Man, Rogosa, and Sharpe (MRS) medium (Difco, Detroit, MI, USA). These strains demonstrated non-hemolytic activity and moderate antibiotic susceptibility to tetracyclines and quinolones. These bacterial strains were identified through 16S rRNA sequencing and were deposited in GenBank (NCBI), accession numbers PV635375 (CNU395) and PV635431 (CNU396). The bacterial strains were prepared with enrichment cultures in MRS medium. The cultures were incubated at 37 °C under anaerobic conditions and shaken for 24 h. Whole bacterial cells were collected by centrifugation and suspended in 10% Difco™ skim milk (Difco, Detroit, MI, USA) to increase stability. Probiotic samples were stored at 4 °C and administered orally at a daily dose of 1 × 10^9^ CFU/mice/day.

### 2.2. Animals, Diets, and Experimental Design

**Figure 1:**
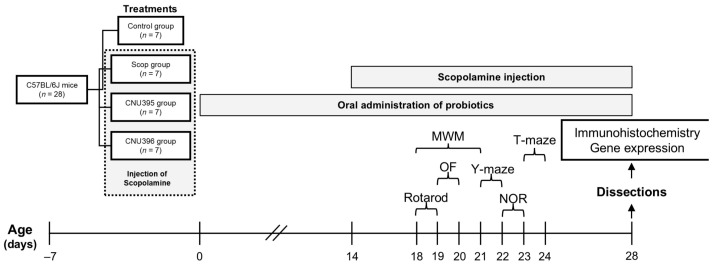
Behavioral experimental schedule for mice injected with scopolamine.

The mice experiments were performed in strict compliance with the recommendations and approval of the Institutional Animal Care and Use Committee of Chonnam National University (Protocol Number: 20170081). Twenty-eight male C57BL/6 mice (8 weeks of age, 21 ± 5 g) were housed under controlled environmental conditions (temperature 22 ± 3 °C, 12 h photoperiod, and 50 ± 5% relative humidity). The mice were provided ad libitum access to a standard diet and sterile water. After acclimating to the environment for 7 days, they were randomly divided into four groups (*n* = 7 per group). In the control group (Control), the mice were intraperitoneally injected with a 0.9% saline solution. The scopolamine-induced cognitive impairment mice (Scop) group were injected with 1 mg/kg/day scopolamine hydrobromide. In the two groups of probiotic-treated mice (CNU395 and CNU396), the mice were orally supplemented with 1 × 10^9^ CFU/mice/day of *L. helveticus* CNU395 and *L. paracasei* CNU396, respectively, for 2 weeks before they were injected with an equal volume of scopolamine hydrobromide (1 mg/kg/day), 18 days before the behavior test. All mice were monitored for 30 min after administration. The experimental procedure is summarized in Figure 1 and designed according to recent investigations [15,16]. The animals were acclimated in a quiet room for 1 h prior to testing to minimize stress. The behavioral variables were automatically recorded and analyzed using the SMART Video Tracking software version 3.0 (Panlab, Harvard Apparatus, Holliston, MA, USA).

### 2.3. Morris Water Maze (MWM)

A MWM procedure was designed in accordance with the method previously described by Morris [17] to evaluate the learning, motor coordination, and spatial memory ability of the mice. Before the test, a water tank with a diameter of 100 cm and a height of 80 cm was prepared and divided into four quadrants. A 50 cm high escape platform with a surface diameter of 9 cm was placed in a fixed quadrant. The water tank was placed in the same position for 4 consecutive days, and the water temperature in the tank was maintained at 22 ± 3 °C. In the acquisition trials, the surface of the platform was 1 cm higher than the water surface. The mice swam freely for 90 s, and if they found the platform, they were allowed to rest for 20 s. If they could not find the platform, they were guided to the platform and allowed to rest for 20 s. On the second trial, the water was raised 1 cm above the platform surface, and the mice were allowed to swim freely for 60 s. If they found the platform, they were allowed to rest on the support for 15 s. These trials were repeated for 4 consecutive days. On the 4th day, after training, the mice were allowed to rest in the cage for 15 min before it entered the test phase. They were gently placed in a random quadrant without the platform, facing the tank wall. They were allowed to swim freely for a maximum of 60 s. The results were evaluated in terms of the following parameters: the average swimming velocity, the time taken to find the holder, the time spent in the quadrant where the holder was placed, the time spent in the opposite quadrant, the number of times the mice swam past the holder, and the distance swam.

### 2.4. Open Field (OF)

An open field (OF) test was conducted to evaluate the exploratory behavior of the mice in a new space, as previously described [18]. The open field measured 40 cm × 40 cm, with a center area of 20 cm × 20 cm. The mice were placed in the center of the field and allowed to explore freely for 10 min. The results were evaluated on the basis of the total distance traveled and the time spent at the edges of the field.

### 2.5. Novel Object Recognition (NOR)

A novel object recognition (NOR) test was performed to assess the ability of the mice to remember and recognize objects, as previously described [19]. On the training day, two objects (A and B) were placed in two symmetrical positions, and the mice were allowed to explore freely for 10 min. After 24 h, two objects (A and C) were placed in the same positions as those on the training day. The mice were allowed, again, to explore freely for 10 min. The results were evaluated on the basis of the new object exploration ratio, which was defined as the ratio of the time spent on the new object (C) to the total time spent on both objects (A and C) by the mice.

### 2.6. Y-Maze

A Y-maze test was conducted to evaluate the spatial memory ability of the mice based on their behavior of exploring new environments, as previously described [20]. The Y-shaped model had three arms (38 cm in length, 8 cm in width, and 13 cm in height, with a 120° spacing). The mice were randomly placed in one arm, facing the wall, and allowed to explore freely for 10 min. The mice with good memory abilities tended to alternate between the three arms, a behavior known as an alternate triad. The starting time was counted from the moment the mice reached the center position. The results were evaluated in terms of the percentage of spontaneous alternation, which was calculated using the following formula: transition rate = (total number of alternate triads)/(total number of mice arms entered − 2) × 100.

### 2.7. T-Maze

A T-maze test was performed to assess the spatial memory ability of the mice based on their exploratory behavior, as previously described [21]. The T-maze used in the experiment was 13 cm high and 38 cm long for the starting arm, as well as 26 cm long for the two target arms from the center. These arms were designated as the starting arm, the expected arm, and the unexpected arm, respectively. On the training day, the mice were placed in the maze at the end of the starting arm, facing the wall. They were allowed to freely explore the maze for 10 min, with the expected arm blocked. After 24 h, the expected arm was opened, and the mice were allowed to explore the maze freely for 10 min. They were placed in the same manner as in the training phase. The mice with good memory abilities tended to enter the expected arm more frequently. The following parameters were recorded: the total number of entries into the expected and unexpected arms, the number of entries per arm, and the time from the start to the entry of the first arm by the mice. The results were evaluated in terms of the ratio of the expected arm entries to the total number of entries in both arms (expected arm entry ratio).

### 2.8. Rota-Rod

A rotarod test was performed to assess the motor coordination and balance of the mice, following the procedure described in Luyten et al. [22]. In brief, the mice were trained to run on a knurled rod with a diameter of 3 cm (LE8205, Panlab, Harvard Apparatus, Holliston, MA, USA) at a fixed speed of 18 rpm for 3 min in a stimulation room under dim light. If a mouse fell during training, it was placed back on the rod, and the timing continued. After training, the mice were allowed to rest in their cage for 15 min. Then, they were gently returned onto the rod, and the running time was recorded from the moment it stood firmly on the rotating bar until it fell off. The test was repeated four times. Each test lasted a maximum of 60 s, and a rest period of 15 min was set between tests. The results were evaluated in terms of the running time of the mice on the rod.

### 2.9. Tissue Collection and Hematoxylin and Eosin (H&E) Staining

After the behavioral session, the brain tissues of the mice were removed, preserved in 4% formaldehyde at 4 °C, embedded in paraffin, sectioned horizontally at a thickness of 4–10 μm, and fixed on glass slides. The subsequent tissue sections were immersed in xylene and graded alcohol to remove the paraffin and then stained with H&E in accordance with standard procedures.

### 2.10. Immunohistochemical Assay

For immunohistochemical staining, the tissue sections were immersed in xylene and graded alcohol to remove the paraffin. Then, they were stained using the 2-Step Plus Poly-HRP anti-mouse/rabbit IgG detection system (Elabscience Biotechnology, Houston, TX, USA) in accordance with the manufacturer’s protocol. The primary antibody against GFAP (1:300) was used.

### 2.11. RNA Extraction and RT-qPCR

A frozen brain tissue (50–100 mg) was placed in a tube containing 1 mL of TRIzol (ThermoFisher, Waltham, MA, USA) and extracted in accordance with the manufacturer’s protocol. The total RNA quantity was determined using NanoDrop 2000c (Wilmington, DE, USA) and subsequently treated with D9Nase. cDNA was synthesized using the Maxima H Minus first-strand cDNA synthesis kit (ThermoFisher Scientific, Waltham, MA, USA) in accordance with the manufacturer’s instructions. The gene expression of cognitive impairment-related genes was assessed via RT-qPCR by using the corresponding primers (Table S1). GADPH was used as a housekeeping gene to normalize the mRNA expression, which was presented as a 2^−ΔΔCt^ value [23].

### 2.12. Statistical Analysis

The data were presented as the mean ± standard error of the mean (SEM). The analysis of most of the parameters of the behavioral and expression investigation was performed through one-way ANOVA with Tukey’s post hoc test GraphPad Prism (version 9.0). The results with *p*-values of less than 0.05 were considered statistically significant.

## 3. Results and Discussion

### 3.1. Morphological Features

Morphological characteristics such as general appearance, body weight, and organ indices were observed in all groups of mice (Table S2). The body weight of the mice did not significantly differ between the groups (*p* > 0.05). The weight of the organs also did not significantly vary (*p* > 0.05).

### 3.2. Effect of Probiotics on Behavior Changes

**Figure 2:**
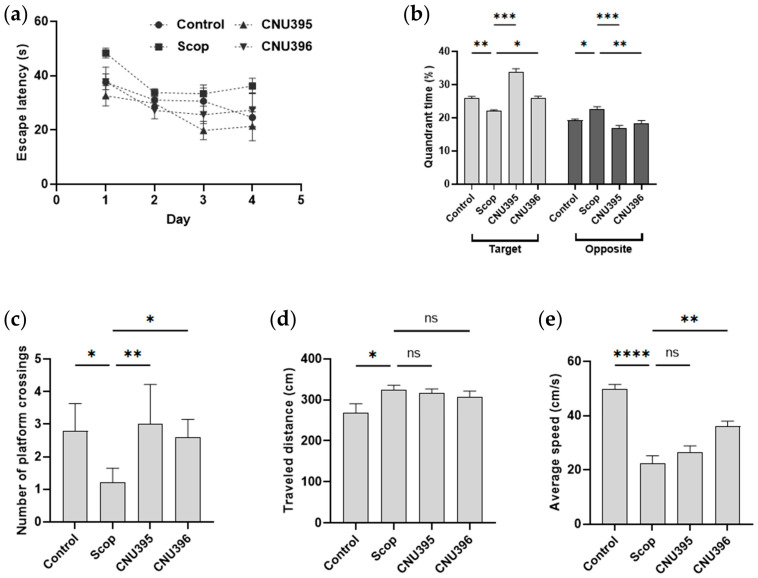
Effect of probiotics on spatial learning and memory of mice in Morris Water Maze trial. The escape latency (**a**), quadrant times (**b**), number of platform crossings (**c**), traveled distance (**d**), and average speed (**e**) of the animals during MWM trial. Data were expressed as mean ± SEM. The *p*-values were determined by one-way ANOVA with Turkey’s post hoc comparisons at * *p* < 0.05; ** *p* < 0.01; *** *p* < 0.001; **** *p* < 0.0001; ns = not significant. Control: 0.9% saline solution, *n* = 7; Scop: 1 mg scopolamine hydrobromide/kg/day, *n* = 7; CNU395: 1 × 10^9^ CFU/mice/day of *L. helveticus* CNU395 and 1 mg scopolamine hydrobromide/kg/day, *n* = 7; CNU396: 1 × 10^9^ CFU/mice/day of *L. paracasei* CNU396 and 1 mg scopolamine hydrobromide/kg/day, *n* = 7.

The cognitive functions of the mice were assessed using the MWM test [24]. Previous studies demonstrated that scopolamine caused cognitive impairment in the MWM test [25,26]. The results showed that the escape latency of the scopolamine-induced cognitive impairment mice increased, while the frequency of the mice swimming through the platform location and their average velocity were significantly lower (*p* < 0.05) than those in the control group (Figure 2). These results indicated that 1 mg/kg/day scopolamine impaired the long-term spatial memory and locomotor abilities of C57BL/6 mice. Additionally, the mice supplemented with probiotics (CNU395 or CNU396) spent significantly more time in the quadrant where the platform was placed (*p* < 0.01 and *p* = 0.024, *F* value = 58.21, respectively) than the scopolamine-induced cognitive impairment mice did. The number of passes through the platform in the two probiotic groups (CNU395 or CNU396) also significantly increased (*p* = 0.086 and *p* = 0.041, *F* value = 5.31) compared with that in the AD group. Therefore, *L. helveticus* CNU395 or *L. paracasei* CNU396 protected against scopolamine-induced cognitive impairment.

**Figure 3:**
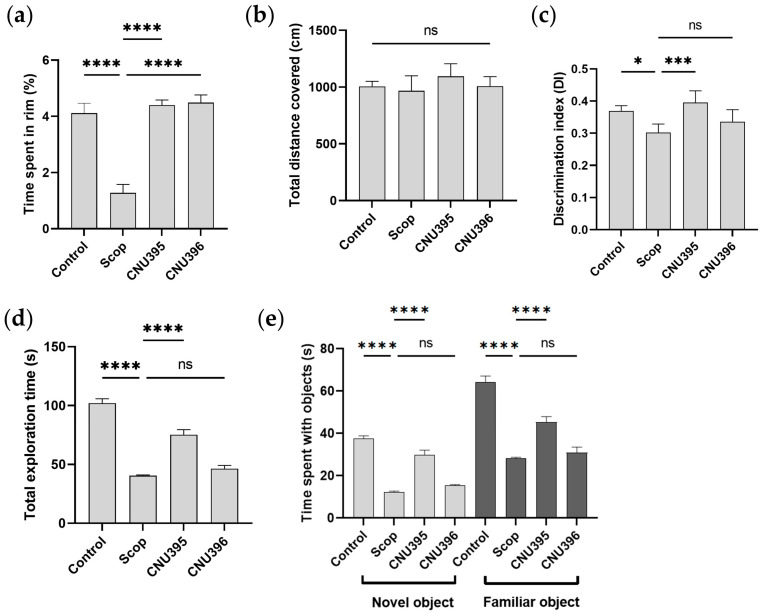
Effect of probiotics on recognition memory of mice in OF and NOR trials. Time spent in rim (**a**) and total distance covered (**b**) of animal in OF test. Discrimination index (**c**), total exploration time (**d**), and time spent with objects (**e**) of the animals in NOR test. Data were expressed as mean ± SEM. The *p*-values were determined by one-way ANOVA with Turkey’s post hoc comparisons at * *p* < 0.05; *** *p* < 0.001; **** *p* < 0.0001; ns = not significant. Control: 0.9% saline solution, *n* = 7; Scop: 1 mg scopolamine hydrobromide/kg/day, *n* = 7; CNU395: 1 × 10^9^ CFU/mice/day of *L. helveticus* CNU395 and 1 mg scopolamine hydrobromide/kg/day, *n* = 7; CNU396: 1 × 10^9^ CFU/mice/day of *L. paracasei* CNU396 and 1 mg scopolamine hydrobromide/kg/day, *n* = 7.

In the OF test, the results showed that significantly more time was spent at the edges of the field (*p* < 0.0001, *F* value = 29.02) by the mice in the scopolamine-induced cognitive impairment group than by the mice in the control group (Figure 3a). Significantly more time was spent in the center by the mice in the probiotic groups than by the mice in the scopolamine-induced cognitive impairment group. Therefore, the probiotics elicited an ameliorative effect against anxiety-like behavior in the scopolamine-induced cognitive impairment mice. In the NOR test, scopolamine significantly reduced the discrimination index, exploration time, and time spent with objects (Figure 3c–e). The mice in the CNU395 probiotic group showed significantly improved memory compared with that of their control counterparts, as evidenced by the longer retention time on new objects (*p* < 0.0001, *F* value = 20.28, Figure 3e). These results suggested that CNU395 might improve spatial short-term memory.

**Figure 4:**
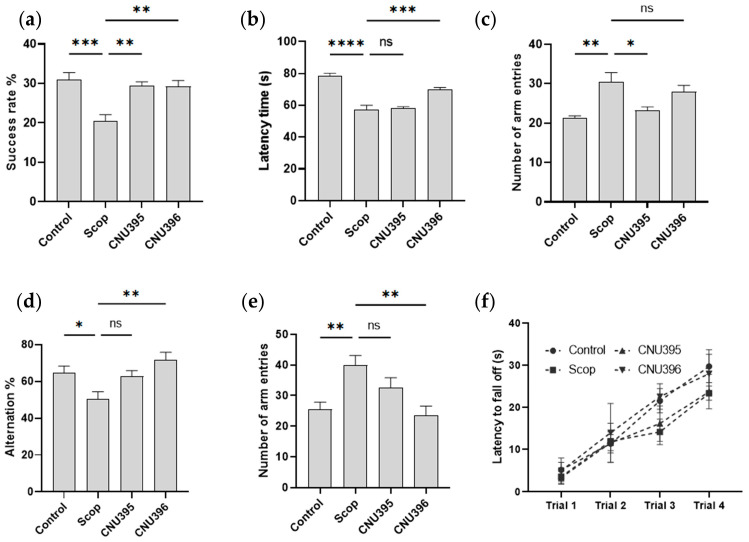
Effect of probiotics on spatial working memory and skill learning ability. Success rate (**a**), latency time (**b**), and number of arm entries (**c**) in T-maze test. Alternation (**d**) and number of arm entries (**e**) in Y-maze test. Latency to fall off (**f**) of mice in rotarod test. Data were expressed as mean ± SEM. The *p*-values were determined by one-way ANOVA with Turkey’s post hoc comparisons at * *p* < 0.05; ** *p* < 0.01; *** *p* < 0.001; **** *p* < 0.0001; ns = not significant. Control: 0.9% saline solution, *n* = 7; Scop: 1 mg scopolamine hydrobromide/kg/day, *n* = 7; CNU395: 1 × 10^9^ CFU/mice/day of *L. helveticus* CNU395 and 1 mg scopolamine hydrobromide/kg/day, *n* = 7; CNU396: 1 × 10^9^ CFU/mice/day of *L. paracasei* CNU396 and 1 mg scopolamine hydrobromide/kg/day, *n* = 7.

Spatial working memory is indicated by the success rate in the T-maze test and the spontaneous alternation percentage in the Y-maze test. In this study, the spatial working memory of the scopolamine-induced cognitive impairment mice was impaired compared with that of the control mice, as evidenced by them passing by the unexpected arm (*p* = 0.0005, *F* value = 10.23, Figure 4a) and a decreased alternation percentage (*p* = 0.0099, *F* value = 5.64 Figure 4d). Conversely, the scopolamine-induced cognitive impairment mice’s decrease in success rate was significantly reversed in the probiotic groups. The alternation percentage markedly increased, suggesting that the working memory of the CNU396 probiotic group was enhanced (*p* = 0.0034). The rotarod test, which detects changes in skill learning ability and coordination, showed that running time did not differ between the groups during the trial (Figure 4f).

In recent years, there has been growing interest in the connection between the gut microbiota and central nervous system diseases [27]. Many studies have emphasized the neuroprotective effects of lactic acid bacterial strains but reports on their role in preventing cognitive impairment in South Korean strains are still limited [28,29]. To the best of our knowledge, this study is the first to investigate the effect of either *L. helveticus* CNU395 and *L. paracasei* CNU396 on scopolamine-induced cognitive impairment mice. Our results provide evidence that the administration of *L. helveticus* CNU395 and *L. paracasei* CNU396 affects cognitive processing. The mice that were treated with probiotics appeared to have the motivation to quickly reach the platform as measured by their escape latency and the number of platform crossings. This result is consistent with previous studies showing that *L. gasseri* NK109 can improve gut microbiota dysbiosis and alleviate cognitive dysfunction, while *L. plantarum* X7022 enhances spatial memory and learning ability in aging mice [30,31]. The average swimming speed of the CNU395 group in the MWM did not significantly differ from the scopolamine-induced cognitive impairment group, indicating that *L. helveticus* CNU395 did not influence general motor abilities. These results need to be further investigated. Interestingly, there was no significant difference in the time spent with objects between the *L. paracasei* CNU396 group and the scopolamine-induced cognitive impairment group. These results highlight the promising potential of *L. helveticus* CNU395 for improving cognitive function. However, further investigation is warranted to explore its precise mechanisms and broader therapeutic applications.

### 3.3. Effect of Probiotics on Morphological Changes

**Figure 5:**
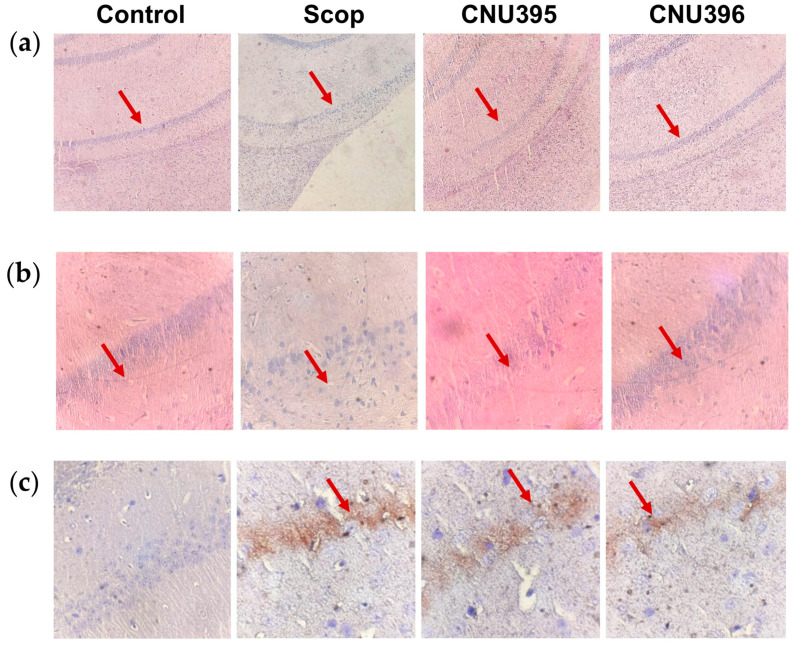
Effect of probiotics on histological change in hippocampus. H&E staining at 10× (**a**) and 40× (**b**). Protein expression of GFAP by immunohistochemistry at 40× (**c**). Red arows indicate neurons in hippocampal CA1 area. Control: 0.9% saline solution; Scop: 1 mg scopolamine hydrobromide/kg/day; CNU395: 1 × 10^9^ CFU/mice/day of *L. helveticus* CNU395 and 1 mg scopolamine hydrobromide/kg/day; CNU396: 1 × 10^9^ CFU/mice/day of *L. paracasei* CNU396 and 1 mg scopolamine hydrobromide/kg/day.

H&E staining and immunohistochemistry were performed to further verify the morphological changes in the cells in the hippocampus (Figure 5). The pyramidal cells in the CA1 region of the hippocampus of the control mice exhibited uniform morphology. The number of pyramidal neurons was significantly reduced in the scopolamine-induced cognitive impairment mice. Additionally, the neurons displayed abnormalities such as shrunken nuclei, uneven nuclear membranes, and degenerated mitochondria. This neuronal damage was alleviated by probiotic treatment. GFAP, an intermediate filament protein in astroglial cells, maintains the shape and mechanical strength of these cells [32]. Astroglial cells may exhibit hypertrophy in neurodegenerative diseases because of GFAP overexpression [33,34,35]. Similarly to previous studies, the present study revealed that the number of GFAP-positive cells in the hippocampus of the scopolamine-induced cognitive impairment mice increased [36]. The significant reduction in GFAP expression by either *L. helveticus* CNU395 or *L. paracasei* CNU396 suggests that these strains mitigated astrocyte activation, protected astrocytes from scopolamine toxicity, and stabilized the hippocampal structure [37]. Combined with the behavioral test results, *L. helveticus* CNU395 and *L. paracasei* CNU396 were shown to alleviate scopolamine-induced damage to learning and memory in mice, preserving normal brain function. Although the relationship between probiotics and GFAP expression is not clear, existing studies suggest that microbial product exposure in the CNS may play a role in cognitive impairment [38,39]. Recent research on fecal microbiota transplantation (FMT) and short-chain fatty acid (SCFA) supplementation indicates that restoration of the gut–brain barrier integrity and reduced neuroinflammation has been associated with the amelioration of pathological increases in GFAP expression in diseased animals [40].

### 3.4. Regulatory Effects of Probiotics on Neuroinflammation

**Figure 6:**
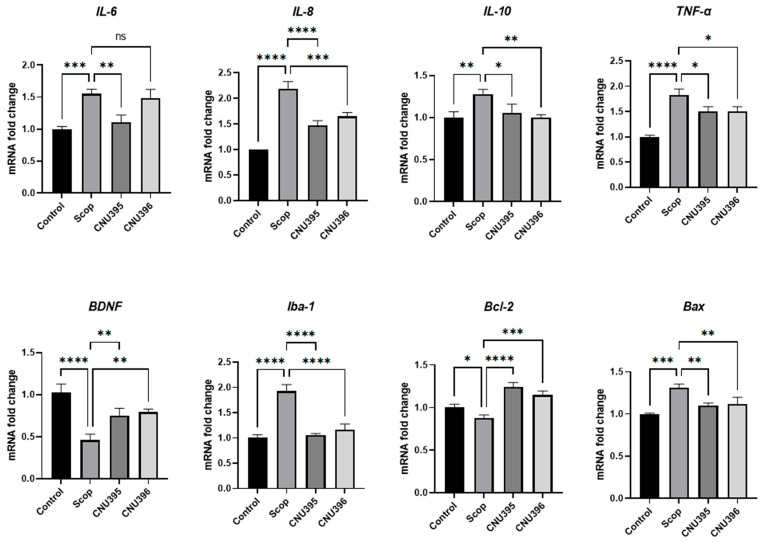
Effect of probiotics on expression of relative genes. Data were expressed as mean ± SEM. The *p*-values were determined by one-way ANOVA with Turkey’s post hoc comparisons at * *p* < 0.05; ** *p* < 0.01; *** *p* < 0.001; **** *p* < 0.0001; ns = not significant. Control: 0.9% saline solution, *n* = 7; Scop: 1 mg scopolamine hydrobromide/kg/day, *n* = 7; CNU395: 1 × 10^9^ CFU/mice/day of *L. helveticus* CNU395 and 1 mg scopolamine hydrobromide/kg/day, *n* = 7; CNU396: 1 × 10^9^ CFU/mice/day of *L. paracasei* CNU396 and 1 mg scopolamine hydrobromide/kg/day, *n* = 7.

To explore the underlying mechanism, we examined the expression of eight targets of pro-inflammatory cytokines, namely, interleukin 6 (*IL-6*), *IL-10*, *IL-8*, tumor necrosis factor-alpha (*TNF-α*), *BDNF*, ionized calcium-binding adaptor molecule 1 (*Iba-1*), B-cell lymphoma protein 2 (*Bcl-2*)-associated X (*Bax*), and *Bcl-2*. *IL-6*, *IL-10*, *IL-8*, *TNF-α*, *Iba-1*, and *Bax* were significantly upregulated, while *BDNF* and *Bcl-2* were significantly downregulated in scopolamine-induced cognitive impairment mice compared with those in the control group (Figure 6). Dietary supplementation with probiotics significantly decreased the expression of *IL-6*, *IL-10*, *IL-8*, and *TNF-α* in the CNU395 group. A similar trend was observed in the CNU396 group, but no significant effect on *IL-6* was detected (*p* = 0.8520, *F* value = 23.91). These results suggested the beneficial role of probiotics in preventing the progression of cognitive impairments though this effect varied among probiotic strains.

The activated microglia act as macrophages to clear extracellular pathogenic Aβ plaques [41]. Microglia are often classified into classical microglia (M1) and alternative microglia (M2) based on phenotype [42]. When activated, M1 microglia secrete pro-inflammatory factors such as TNF-α and IL-6 [43]. Conversely, the activation of M2 microglia promotes the repair of damaged neural tissues by releasing anti-inflammatory factors such as IL-10 and neurotrophic factors such as BDNF. In the progression of AD, M2 microglia are activated during the early stages, whereas overstimulation in advanced stages leads to a rise in the inflammatory markers associated with M1 microglia [44]. Additionally, previous studies demonstrated that the expression levels of pro- and anti-inflammatory molecules increased in AD cases [45,46]. IL-6 has been considered a potential key mediator of gut–brain communication [47]. Indeed, elevated IL-6 levels have been linked to disruptions in the gut microbiota, which may contribute to cognitive and neurological disorders [48]. Previous studies showed that *L. helveticus* HY7801 suppressed E2-mediated prolactin increases and pro-inflammatory IL-6 cytokine secretion [49]. Based on the results obtained, our study revealed that the administration of CNU395 decreased *IL-6* expression and was more effective than CNU396.

IL-8, another factor secreted by microglia, participates in the inflammatory process of neurodegenerative diseases. IL-8 expression can exacerbate the inflammatory response in AD brains by enhancing the activity of Aβ plaques [50]. Additionally, scopolamine can cause neuroinflammation, one of the major hallmarks in the progression of AD [51]. The present study confirmed that probiotic administration could decrease the expression of pro-inflammatory cytokines, suggesting that probiotics could reduce inflammation in a mice brain.

BDNF maintains synaptic plasticity in learning and memory [52]. The occurrence of pathogenic Aβ plaques is associated with reduced BDNF activation [53]. Recently, we reported that BDNF was activated in scopolamine-induced cognitive impairment mice treated with CNU395 or CNU396 (Figure 6). Our study suggested that the beneficial effects of probiotics on oxidative stress and plasticity maintenance might be significantly achieved by regulating BDNF signaling in scopolamine-induced cognitive impairment mice. In addition, Iba-1 is a protein that binds to microglia and can be used to mark microglial activity in cells [54]. It is increased in neurodegenerative conditions, including AD [55,56]. CNU395 or CNU396 significantly decreased the expression levels of *Iba-1* in the cortex and hippocampus.

AD is characterized by increased phosphorylation of the tau protein, leading to the dissociation of tau from microtubules, followed by microtubule destabilization and tau protein aggregation; consequently, intracellular neurofibrillary tangles form. The gradual accumulation of these tangles leads to apoptosis [44]. Abnormal apoptosis is considered one of the factors causing neurodegenerative diseases, including AD. This process is mainly regulated by two proteins, Bax and Bcl-2; specifically, it is stimulated by Bax but inhibited by Bcl-2 [57]. In neurodegenerative diseases, the expression of Bax increases, while the expression of Bcl-2 decreases [58]. Our study also found a decrease in *Bax* expression and an upregulation of *Bcl-2* expression in CNU395- and CNU396-treated mice (Figure 6). These results are in line with the meta-analysis of Tripathi et al., which highlights the role of probiotics in influencing oxidative stress and inflammatory markers in mild cognitive impairment and Alzheimer’s disease, particularly *Lactobacillus johnsonii*, *Bifidobacterium infantis*, and *Bifidobacterium breve* [59]. Thus, this connection underscores the potential of novel *L. helveticus* CNU395 probiotic strains and their intricate relationship with the gut microbiota, inflammation, and behavioral effects.

### 3.5. Regulatory Effects of Probiotics on MAPK and NF-κB Signaling Pathways

**Figure 7:**
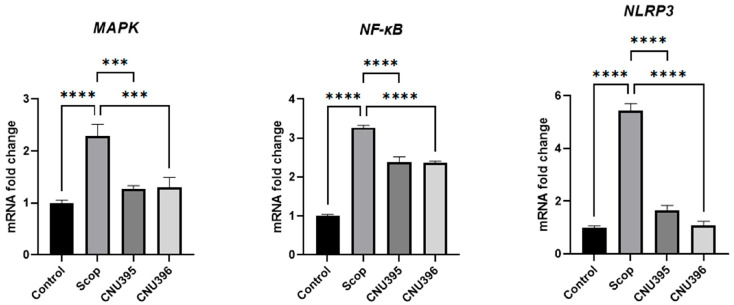
Effect of probiotics on expression of p38 MAPK and NF-κB. Data were expressed as mean ± SEM. The *p*-values were determined by one-way ANOVA with Turkey’s post hoc comparisons at *** *p* < 0.001; **** *p* < 0.0001. Control: 0.9% saline solution, *n* = 7; Scop: 1 mg scopolamine hydrobromide/kg/day, *n* = 7; CNU395: 1 × 10^9^ CFU/mice/day of *L. helveticus* CNU395 and 1 mg scopolamine hydrobromide/kg/day, *n* = 7; CNU396: 1 × 10^9^ CFU/mice/day of *L. paracasei* CNU396 and 1 mg scopolamine hydrobromide/kg/day, *n* = 7.

Scopolamine activates NLRP3 activity via both mitogen-activated protein kinase (MAPK) and nuclear factor-kappa B (NF-κB) signaling pathways during scopolamine-induced neuroinflammatory responses [60]. To investigate whether probiotics regulated the activation of MAPK and NF-κB signaling pathways in scopolamine-induced cognitive impairment mice, the expression of *MAPK*, *NF-κB*, and *NLRP3* were examined by RT-qPCR. As shown in Figure 7, *L. helveticus* CNU395 and *L. paracasei* CNU396 significantly decreased the expression of *MAPK*, *NF-κB*, *and NLRP3* in scopolamine-induced cognitive impairment mice. The p38 MAPK and NF-κB p65 signaling pathway has been suggested to be involved in the progression of neurodegenerative diseases [61]. The production of pro-inflammatory cytokines, including IL-1β, TNF-α, and IL-6, is mainly via the MAPK signaling pathway [62]. According to previous reports it was hypothesized that the probiotics strains may regulate both the MAPK and NF-κB signaling pathways to prevent inflammation-associated disorders. The significant reduction in MAPK may be attributed to short-chain fatty acids produced by *Lactobacillus*, which regulate IL-6 expression [63]. *Lactobacillus* strains, specifically the *L. helveticus* KLDS1.8701 down-regulated translocation of NF-κB p65 and suppressed p38 MAPK signaling pathway activation in the LPS-induced RAW264.7 cells [64]. Evidence has demonstrated that *Lactobacillus paracasei* GMNL-32 mitigates liver inflammation by reducing the activity of MMP-9 and the expressions of CRP, iNOS, IL-1*β*, IL-6, and TNF-*α* proteins by inhibiting MAPK/NF-*κ*B inflammatory signaling [65]. Similarly, our results shown that *L. helveticus* CNU395 and *L. paracasei* CNU396 suppress scopolamine-stimulated *NLRP3* inflammasome activation by inhibiting the activation of both *MAPK* and *NK-κB* signaling in vivo, indicating that *L. helveticus* CNU395 and *L. paracasei* CNU396 has potential in attenuating microglia-induced inflammation.

## 4. Conclusions

This study demonstrated that probiotic supplementation by either CNU395 or CNU396 attenuated the degree of cognitive dysfunction in scopolamine-induced cognitive impairment mice. It could positively alleviate neuroinflammation in the brains of the mice by inhibiting the expression of inflammatory precursors (*IL-6*, *IL-8*, *IL-10*, *and TNF-α*) by inhibiting the *MAPK* and *NF-κB* inflammatory pathways, and protecting neuronal survival and proliferation via *BDNF*, *Bax*, *and Bcl-2*. Because of the regulatory effects on the gastrointestinal–neural axis, CNU395 might be a promising and safe nutritional supplement for regular use to prevent and limit cognitive dysfunction. We also show that these effects on behavior change across trials, likely due to differences among bacterial strains. However, we have very sparse metabolomics and gut microbiome data, which will be important to better understand the molecular mechanism of gastrointestinal–neural axis. In future studies, metabolites that transmit signals from the gut to the nervous system and participate in the therapeutic activity of probiotics should be identified. The modulation of the gut microbiota during probiotic administration should also be investigated to ensure microbial balance.

## Data Availability

The original contributions presented in the study are included in the article/Supplementary Materials; further inquiries can be directed at the corresponding author.

## Supplementary Materials

The following supporting information can be downloaded at: https://www.mdpi.com/article/10.3390/microorganisms13081714/s1, Table S1: List of primers; Table S2: The average body weight and organ index of mice in each experimental group.

## Abbreviations

The following abbreviations are used in this manuscript:

AD	Alzheimer’s disease
Ach	Acetylcholine
AChE	Acetylcholinesterase
Aβ	Amyloid beta
Bax	B-cell lymphoma protein 2-associated X
Bcl-2	B-cell lymphoma protein 2
BDNF	Brain-derived neurotrophic factor
H&E	Hematoxylin and Eosin
Iba-1	Ionized calcium-binding adaptor molecule 1
IL	Interleukin
MAPK	Mitogen-activated protein kinase
MRS	de Man, Rogosa, and Sharpe
MWM	Morris water maze
NF-κB	Nuclear factor-kappa B
NOR	Novel object recognition
OF	Open field
TNF-α	Tumor necrosis factor-alpha
