# Supplementary material for: Lactobacillus helveticus CNU395 and L. paracasei CNU396 Alleviate Cognition in Scopolamine-Induced Cognitive Impairment Mice
Source: Microorganisms. 2025 Jul 22;13(8):1714. doi: 10.3390/microorganisms13081714 (PMC12388029; doi:10.3390/microorganisms13081714)
Supplement: Supplementary file 1 [file microorganisms-13-01714-s001.zip › microorganisms-3734594-supplementary.pdf]

Table S1. List of primers

Gene	Primer	Sequence
bdnf	Forward	ATCCACTGAGCAAAGCCGAA
	Reverse	CCTGGTGGAACATTGTGGT
bax	Forward	TGCAGAGGATGATTGCTGAC
	Reverse	GATCAGCTCGGGCACTTTAG
bcl-2	Forward	AGTACCTGAACCGGCATCTG
	Reverse	GCTGAGCAGGGTCTTCAGAG
iba-1	Forward	TGAGGAGATTTCAACAGAAGCTGA
	Reverse	CCTCAGACGCTGGTTGTCTT
il-6	Forward	GAGGATACCACTCCCAACAGACC
	Reverse	AAGTGCATCATCGTTGTTCATACA
il-8	Forward	CTAGGCATCTTCGTCCGTCC
	Reverse	TTCACCCATGGAGCATCAGG
il-10	Forward	TAAGGGTTACTTGGGTTGCCA
	Reverse	GAGAAATCGATGACAGCGCC
tnf-α	Forward	GTCCCCAAAGGGATGAGAAGT
	Reverse	TGGTTTGCTACGACGTGGG
mapk	Forward	ATGGTGCAGGAAAACAGGAC
	Reverse	CGTCTCTCCCTTTGTTCAGC
nf-κb	Forward	TGACTGTGGAGCTGAAGTGG
	Reverse	GCCTTTAGAAGGAGGCGAGT
nlrp3	Forward	ATGCTGCTTCGACATCTCCT
	Reverse	AACCAATGCGAGATCCTGAC
gadph	Forward	GCATCTTCTTGTGCAGTGCC
	Reverse	GGTCAGCAAGACCTGCGTA

Table S2. The average body weight and organ index of mice in each experimental group.

Group	Body weight (g)	Brain (g)	Kidney (g)	(Liver (g)	Spleen (g)
Control	18.8 ± 1.63	0.39 ± 0.067	0.22 ± 0.065	0.85 ± 0.121	0.06 ± 0.030
Scop	19.5 ± 1.63	0.39 ± 0.027	0.23 ± 0.012	0.83 ± 0.031	0.04 ± 0.004
CNU395	20.6 ± 0.49	0.40 ± 0.012	0.25 ± 0.015	0.83 ± 0.105	0.04 ± 0.003
CNU396	19.2 ± 0.88	0.38 ± 0.016	0.23 ± 0.011	0.85 ± 0.027	0.04 ± 0.006
